# Evaluation of Monoexponential, Stretched‐Exponential and Intravoxel Incoherent Motion MRI Diffusion Models in Early Response Monitoring to Neoadjuvant Chemotherapy in Patients With Breast Cancer—A Preliminary Study

**DOI:** 10.1002/jmri.28113

**Published:** 2022-02-14

**Authors:** Zyad M. Almutlaq, Daniel J. Wilson, Sarah E. Bacon, Nisha Sharma, Samuel Stephens, Tatendashe Dondo, David L. Buckley

**Affiliations:** ^1^ Biomedical Imaging University of Leeds Leeds UK; ^2^ Radiological Sciences Department, College of Applied Medical Sciences King Saud bin Abdulaziz University for Health Sciences Riyadh Saudi Arabia; ^3^ Department of Medical Physics & Engineering Leeds Teaching Hospitals NHS Trust Leeds UK; ^4^ Department of Radiology Leeds Teaching Hospitals NHS Trust Leeds UK; ^5^ Clinical and Population Sciences Department, Leeds Institute of Cardiovascular and Metabolic Medicine University of Leeds Leeds UK

**Keywords:** breast cancer, diffusion‐weighted MRI, neoadjuvant chemotherapy, quantitative evaluation, imaging biomarkers

## Abstract

**Background:**

There has been a growing interest in exploring the applications of stretched‐exponential (SEM) and intravoxel incoherent motion (IVIM) models of diffusion‐weighted imaging (DWI) in breast imaging, with the focus on differentiation of breast lesions. However, the use of SEM and IVIM models to predict early response to neoadjuvant chemotherapy (NACT) has received less attention.

**Purpose:**

To investigate the value of monoexponential, SEM, and IVIM models to predict early response to NACT in patients with primary breast cancer.

**Study Type:**

Prospective.

**Population:**

Thirty‐seven patients with primary breast cancer (aged 46 ± 11 years) due to undergo NACT.

**Field Strength/Sequences:**

A 1.5‐T MR scanner, T_1_ ‐weighted three‐dimensional spoiled gradient‐echo, two‐dimensional single‐shot spin‐echo echo‐planar imaging sequence (DWI) at six *b*‐values (0–800 s mm^−2^).

**Assessment:**

Tumor volume, apparent diffusion coefficient, tissue diffusion (*D*_*t*_), pseudo‐diffusion coefficient (*D*_*p*_), perfusion fraction (*f*), distributed diffusion coefficient, and alpha (*α*) were extracted, following volumetric sampling of the tumors, at three time‐points: pretreatment, post one and three cycles of NACT.

**Statistical Tests:**

Mann–Whitney test, receiver operating characteristic (ROC) curve. Statistical significance level was *P* < 0.05.

**Results:**

Following NACT, 17 patients were determined to be pathological responders and 20 nonresponders. Tumor volume was significantly larger in nonresponders at each MRI time‐point and demonstrated reasonable performance in predicting response (area under the ROC curve [AUC] = 0.83–0.87). No significant differences between groups were found in the diffusion coefficients at each time‐point (*P* = 0.09–1). The parameters *α* (SEM), *f*, and *f* × *D*_*p*_ (IVIM) were able to differentiate between response groups after one cycle of NACT (AUC = 0.73, 0.72, and 0.74, respectively).

**Conclusion:**

Diffusion coefficients derived from the monoexponential, SEM, and IVIM models did not predict pathological response. However, the IVIM‐derived parameters *f* and *f* × *D*_*p*_ and the SEM‐derived parameter *α* were able to predict response to NACT in breast cancer patients following one cycle of NACT.

**Level of Evidence:**

2

**Technical Efficacy Stage:**

2

Neoadjuvant chemotherapy (NACT) is a widely used treatment approach in patients with breast cancer to reduce tumor size and increase the chances of breast‐conserving surgery.^1^, ^2^ However, it is associated with considerable toxicity. Identifying nonresponders before or at an early stage of treatment is valuable, allowing clinicians to change the NACT regimen or proceed to surgery without delay, avoiding toxic side effects of NACT and tumor progression while maintaining the cost‐effectiveness of the treatment plan.^3^

Treatment response to NACT can be evaluated through clinical examination and imaging results, such as mammography, ultrasonography, and MRI. However, these imaging techniques are usually limited to evaluating morphological changes.^4^, ^5^ Physiologic and microstructural changes often precede morphologic changes.^6^ Therefore, functional imaging techniques may allow a therapeutic response evaluation at an earlier treatment stage.

Diffusion‐weighted MRI (DWI) measures the random, Brownian, movement of water in tissue. The diffusivity of water molecules is affected by changes in the tissue microstructure, including tissue cellularity and membrane integrity.^7^ The apparent diffusion coefficient (ADC) can be calculated and used to quantify water‐molecule diffusion in tissue. Therapy‐induced cell lysis, apoptosis, or necrosis leads to less restriction and increased ADC values.^8^ The link between ADC and tumor cell density makes it useful for monitoring cytotoxic responses.^9^ However, intertumoral structural heterogeneity may lead to heterogeneity of water diffusion in the tumor resulting in non‐monoexponential diffusion.^10^ Moreover, blood within the capillary network as well as tissue diffusion contributes to the ADC value, which may affect its accuracy in describing diffusion.^11^

Bennett et al introduced the stretched‐exponential model (SEM) to assess intravoxel heterogeneity of diffusion by measuring the distributed diffusion coefficient (DDC) and the diffusion heterogeneity index (*α*), which ranges from 0 to 1. A low *α* index indicates a high degree of intravoxel diffusion heterogeneity, demonstrated as multiexponential signal decay, whereas a high *α* index (close to 1) indicates a low degree of diffusion heterogeneity, suggesting monoexponential diffusion signal decay.^12^ Accumulating evidence reveals that SEM is useful for evaluating breast and other tumors; however, the application of SEM in assessing the breast cancer response to NACT is still limited.^13^, ^14^, ^15^, ^16^, ^17^, ^18^, ^19^, ^20^

The intravoxel incoherent motion (IVIM) model separates the effects of perfusion from tissue diffusion using multiple *b*‐values (a measure of the diffusion sensitivity of DWI) and a biexponential analysis, thus enabling the measurement of diffusion‐related parameter *D*_*t*_ (reflecting tissue diffusion) and perfusion‐related parameters, including *D*_*p*_ (reflecting the pseudo‐diffusion coefficient), *f* (reflecting the perfused fraction), and the product of *f* and *D*_*p*_ (reflecting microvascular blood flow).^11^, ^21^ Several studies have shown the potential value of the IVIM model in differentiating between benign and malignant breast tumors^14^, ^22^, ^23^; however, few studies have investigated its ability to assess the response of breast cancer to NACT.^20^, ^24^

Two preliminary studies have found that, after two or three cycles of NACT, parameters of the SEM and IVIM models obtained from a single freehand region of interest (ROI) drawn on the imaging slice with the largest tumor dimension may predict breast cancer response to NACT.^20^, ^24^ Authors of both studies recommend further investigations at earlier treatment time points (i.e., after one cycle). Furthermore, volumetric sampling has been recommended by the international breast DWI working group when evaluating tumor response.^25^

The aim of this study was to investigate the value of monoexponential, SEM, and IVIM models obtained pretreatment and after one and three cycles of NACT, using volumetric sampling, to assess early breast cancer response to NACT.

## Materials and Methods

### Patients

A local research ethics committee approved the study, and written informed consent was obtained from each subject. The patient inclusion criteria were 1) 18 years of age and over; 2) pathological confirmation of an invasive breast cancer via core needle biopsy; and 3) planned to undergo NACT. Patients were ineligible if they received previous treatment for breast cancer (eg, radiotherapy or chemotherapy) or had recurrent breast cancer, impaired kidney function, or contraindications to MRI. Patients recruited were treated with a standardized protocol of at least six cycles of NACT, where all patients received epirubicin with cyclophosphamide for the first three cycles, followed by three cycles of docetaxel. In patients with human epidermal growth factor receptor 2 (HER2) positive tumors, docetaxel was accompanied by trastuzumab, and in some (more recent) cases pertuzumab.

### Magnetic Resonance Imaging

All patients were imaged on a 1.5‐T MRI scanner (Aera; Siemens) using a 16‐channel bilateral breast coil (Sentinelle; Siemens) with the patient in a head‐first prone position. The MRI protocol included axial T_1_‐weighted three‐dimensional spoiled gradient echo (FLASH), axial T_2_‐weighted turbo spin‐echo, DWI, and dynamic contrast‐enhanced (DCE) series. Axial DWI was performed before DCE‐MRI using a spectral attenuated inversion‐recovery fat‐suppressed, two‐dimensional single‐shot spin echo–echo planar imaging sequence at six *b*‐values (0, 50, 100, 200, 400, and 800 s mm^−2^) with the following parameters: repetition time/echo time: 7200/59 msec, field of view (FoV): 340 × 136 mm, matrix size: 280 × 116, slice thickness: 4 mm, and parallel imaging (generalized autocalibrating partially parallel acquisitions; GRAPPA) factor: 2. The acquisition time of the DWI sequence was 5 minutes, 31 seconds. ADC maps were created by the scanner software immediately following DWI acquisition.

DCE‐MRI was performed using a fat‐suppressed T_1_‐weighted three‐dimensional FLASH sequence (repetition time/echo time: 4.1/1.2 msec, FoV: 340 × 340 × 180 mm^3^, matrix size: 384 × 384 × 128, flip angle: 10°, parallel imaging (GRAPPA) factor: 3, and acquisition time: 36 seconds) to acquire images before and approximately 2 minutes after an intravenous bolus injection of 0.1 mmol kg^−1^ Gd‐DOTA (Dotarem, Guerbet Laboratories). Subtraction images were generated for each patient by subtracting the precontrast from the postcontrast images to enhance tumor visibility.

MRI was performed at four time points: before NACT (pretreatment) and after one, three (mid‐treatment), and six cycles of NACT (images acquired post six cycles were not included in this analysis).

### Image Analysis

Using commercial software (NUMARIS/4, Syngo MR B17, Siemens), DCE subtraction images were registered (rigid body) to the corresponding DWIs. The registered DCE subtraction images and original DWI data were exported into MATLAB (MathWorks).

**FIGURE 1:**
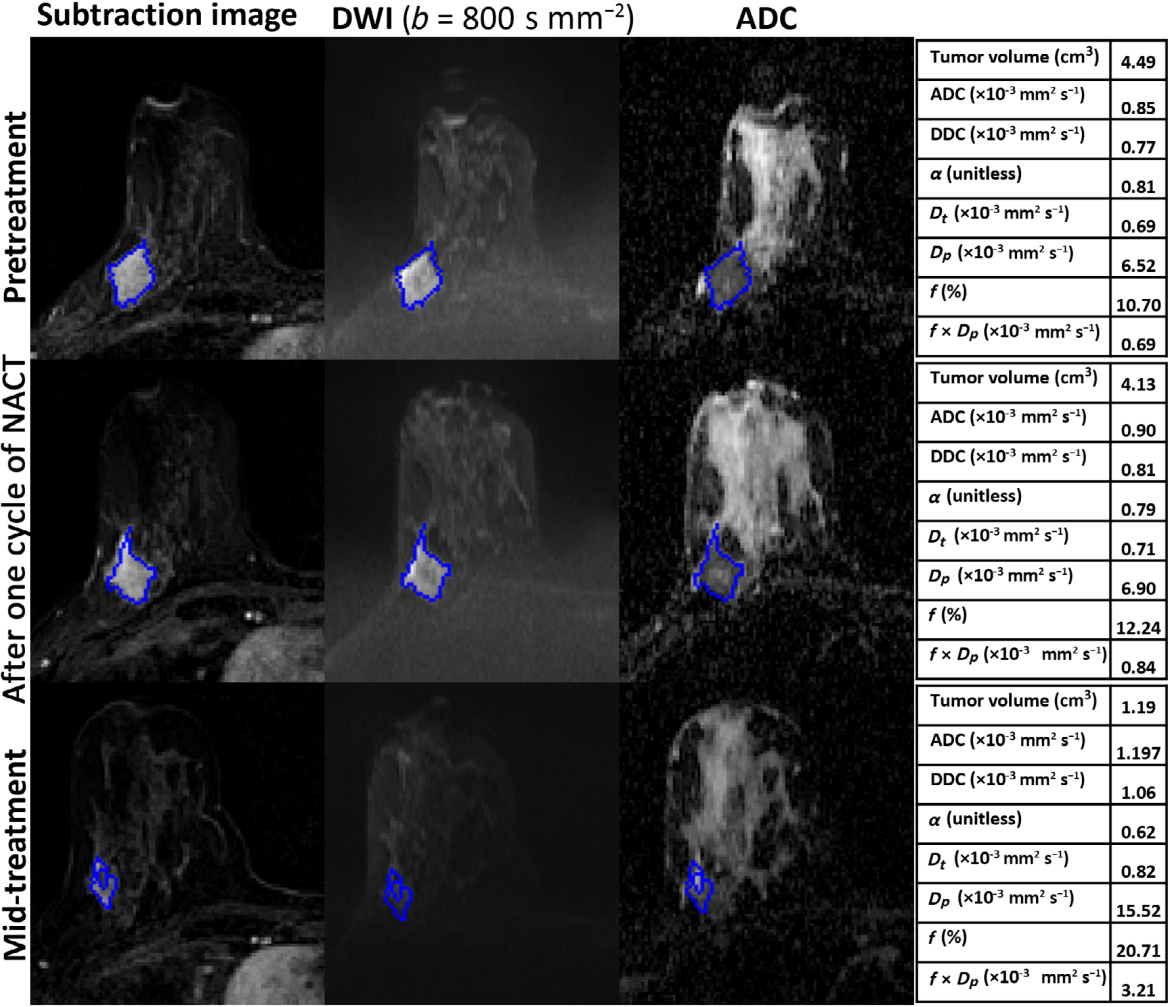
MRI scans of a 39‐year‐old woman with invasive ductal carcinoma who was a nonresponder (residual cancer burden [RCB]‐II). Each row includes images acquired pretreatment, after one cycle of neoadjuvant chemotherapy (NACT), and at mid‐treatment. The seeded region of interest (ROI) for the given slice is shown in blue. The tables represent the parameter estimates of monoexponential, stretched‐exponential model (SEM) and intravoxel incoherent motion (IVIM) model at each time‐point.

For each patient, the location of the largest tumor was identified in the DCE images of the pretreatment MRI and confirmed by a breast radiologist (N.S) with 11 years of experience in breast MRI. Then, a radiographer (Z.M; 2 years' experience in breast MRI analysis) used an in‐house program developed in MATLAB to seed the tumor and generate a whole‐volume ROI using a three‐dimensional‐region growing algorithm based on a threshold signal intensity (SI) of the enhanced lesion in the DCE subtraction images. Obvious necrotic areas were avoided manually. The tumor volume was calculated from the sum of all enhanced tumor voxels. This ROI was transferred to the corresponding DWI, and the average SI value for every *b*‐value was extracted^26^ (Fig. 1).

**FIGURE 2:**
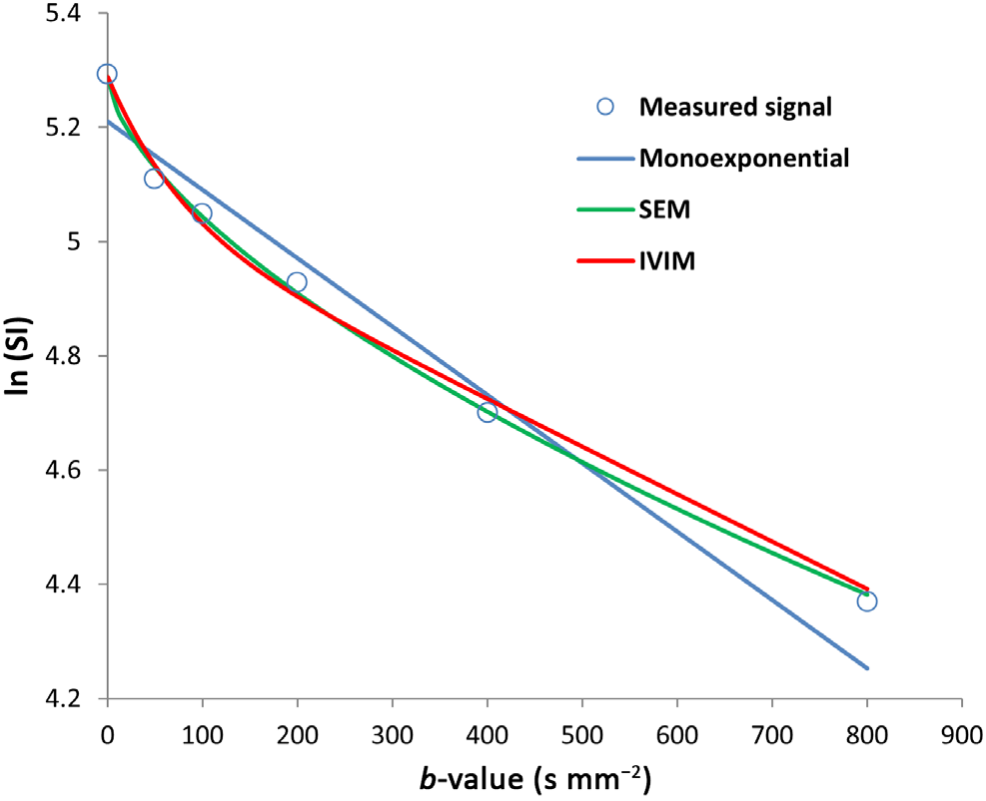
The measured diffusion‐weighted imaging (DWI) signals and best‐fit curves of the tumor of the nonresponder patient in Fig. 1 at mid‐treatment.

The monoexponential, SEM, and IVIM models were fitted to the average SI vs. *b*‐value data using a nonlinear least‐squares approach (Fig. 2). Parameters of the monoexponential (ADC), SEM (DDC, *α*), and IVIM (*D*_*t*_, *D*_*p*_, and *f*) models were estimated using the entire range of *b*‐values.^27^ Electronic supplementary material (ESM) (Appendix E1) details the equations used for the three models.

Parameter estimates and tumor volume were obtained and recorded for each patient at each MRI time‐point. In addition, the percentage changes in the parameters and tumor volume compared to pretreatment values were calculated for each patient as:

$$\left[\left({\text{Value}}_{\text{after}\;\mathrm{one}\;\text{cycle}/\text{three cycles}}\right)-\left({\text{Value}}_{\text{pretreatment}}\right)\right]/\left({\text{Value}}_{\text{pretreatment}}\right)\times 100\%.$$

Interobserver variability in the tumor volume measurements was assessed with a second reader repeating the ROI seeding exercise (S.S; less than 1 year' experience in breast MRI analysis). All steps were conducted blinded to the patients' pathological responses, which were evaluated after surgery.

### Pathological Response Evaluation

As previously reported,^28^ the tumor's response was assessed by a pathologist who derived a residual cancer burden (RCB) index by dissecting and histologically examining the resected surgical specimen after completion of all cycles of NACT. RCB can be separated into four classes (RCB‐0 to RCB‐III), in which RCB‐0 denotes a pathologic complete response to NACT (pCR), which is associated with a good prognosis, whereas RCB‐III denotes extensive residual disease, which is associated with a poor prognosis. Patients with an RCB class I have the same 5‐year prognosis as those with RCB class 0.^29^

Patients were divided into two groups: pathological responders (pR), with an RCB class of 0 or I (RCB index ≤ 1.36) and pathological nonresponders (pNR), with an RCB class of II or III (RCB index > 1.36).

### Statistical Analysis

Median and interquartile ranges were used to summarize the DWI model parameters due to the nonnormal distribution of the data. Interobserver agreement in the tumor volume measurements obtained at all three time‐points was analyzed using the intraclass correlation coefficient (ICC) (ICC < 0.5: poor agreement, 0.5 ≤ ICC < 0.75: moderate agreement, 0.75 ≤ ICC < 0.9: good agreement, and 0.9 ≤ ICC: excellent agreement).^30^ Differences in the parameters before NACT (pretreatment), post one cycle, and post three cycles were compared for all patients using Friedman's test with Bonferroni correction (Bonferroni post hoc test). The parameter estimates of pR and pNR were compared using the Mann–Whitney test. The percentage change in the parameter values after one and three cycles of NACT was compared between pR and pNR.

Receiver operating characteristic (ROC) curves were generated to assess the performance of the parameters to predict treatment outcomes, summarized by calculating areas under the ROC curves (AUC) (0.5 ≤ AUC < 0.7: poor accuracy and 0.7 ≤ AUC < 0.9: reasonable accuracy).^31^ All analyses were performed using IBM SPSS (v.25.0). As this study is preliminary, *P*‐values for the predictive tests were presented as raw values and not corrected for multiple comparisons. Thus, a *P*‐value < 0.05 was considered statistically significant.

## Results

Between August 2015 and April 2018, 40 female patients (mean age 46, range 25–69 years) were eligible and recruited to this study. Data analysis was performed on 37 of the 40 patients recruited. Three patients withdrew following their pretreatment study and did not undergo further MRI. According to the RCB assessment following surgery, 17 (46%) patients were classified as pR, whereas 20 (54%) patients were considered pNR. The characteristics of the enrolled patients and tumors are summarized in Table 1. Compared with the pR patients, pNR patients were older (50 years, SD ±9 vs. 42 years, SD ±12) and had a higher proportion of grade‐III tumors [65% (13) vs. 53% (9)].

**TABLE 1:** . Tumor Characteristics of Enrolled Patients

Characteristic	Number
Age, years (mean ± SD)	46.2 ± 10.6
Tumor grade	
II	15
III	22
Tumor histology	
Invasive ductal carcinoma	35
Inflammatory breast cancer	1
Mucinous carcinoma	1
Estrogen receptor status	
Positive (+)	25
Negative (−)	12
Progesterone receptor status	
Positive (+)	16
Negative (−)	19
Not evaluable	2
Human epidermal growth factor 2 status	
Positive (+)	15
Negative (−)	22

Table 2 presents values at pretreatment and post one and three cycles of NACT across the cohort. Pretreatment tumor volume was significantly higher than tumor volume after three cycles of NACT [median (cm^3^): 4.67 and 2.04, respectively]. DDC and *D*_*t*_ pretreatment and after one cycle were significantly lower than those after cycle 3 [median: 0.94, 1, and 1.13 for DDC (×10^−3^ mm^2^ s^−1^), and 0.83, 0.86, and 0.95 for *D*_*t*_ (×10^−3^ mm^2^ s^−1^), respectively]. ADC and *f* were significantly lower pretreatment than after cycle 3 [median: 1 and 1.2 for ADC (×10^−3^ mm^2^ s^−1^), and 12.23 and 15.51 for *f* (%), respectively].

**TABLE 2:** Tumor Volume and Parameters of Monoexponential, SEM, and IVIM Models Pretreatment, After One Cycle of NACT, and at Mid‐treatment

Parameter	Pretreatment (a)	After One Cycle of NACT (b)	After Three Cycles of NACT (c)	*P*	Post hoc**
Tumor volume (cm^3^)	4.67 (2.03, 13.11)	3.06 (1.63, 8.83)	2.04 (0.62, 5.27)	<0.001	a > c
ADC (×10^−3^ mm^2^ s^−1^)	1 (0.9, 1.21)	1.04 (0.98, 1.21)	1.2 (1.05, 1.38)	<0.001	a < c
DDC (×10^−3^ mm^2^ s^−1^)	0.94 (0.82, 1.15)	1 (0.9, 1.17)	1.13 (0.99, 1.35)	<0.001	a < c, b < c
*α* (unitless)	0.83 (0.79, 0.87)	0.85 (0.8, 0.9)	0.84 (0.8, 0.89)	0.41	‐
*D*_*t*_ (×10^−3^ mm^2^ s^−1^)	0.83 (0.73, 0.94)	0.86 (0.79, 1.01)	0.95 (0.85, 1.14)	<0.001	a < c, b < c
*D*_*p*_ (×10^−3^ mm^2^ s^−1^)	6.53 (5.3, 7.44)	6.01 (4.68, 7.31)	5.87 (4.59, 7.77)	0.68	‐
*f* (%)	12.23 (10.07, 15.79)	14.16 (11.47, 17.13)	15.51 (13.56, 17.87)	<0.001	a < c
*f* × *D*_*p*_ (×10^−3^ mm^2^ s^−1^)	0.88 (0.69, 1.05)	0.83 (0.57, 1.13)	0.97 (0.7, 1.34)	0.28	‐

Data represented by medians (interquartile ranges). *P* value for a difference between the three visits was found using Friedman's nonparametric test. Pairwise comparisons** (Bonferroni‐corrected) significance at the 0.05 level.

SEM = stretched‐exponential model; IVIM = intravoxel incoherent motion; NACT = neoadjuvant chemotherapy; ADC = apparent diffusion coefficient; DDC = distributed diffusion coefficient; *α* = diffusion heterogeneity index; *D*_*t*_ = tissue diffusion; *D*_*p*_ = pseudo‐diffusion coefficient; *f* = perfusion fraction.

**FIGURE 3:**
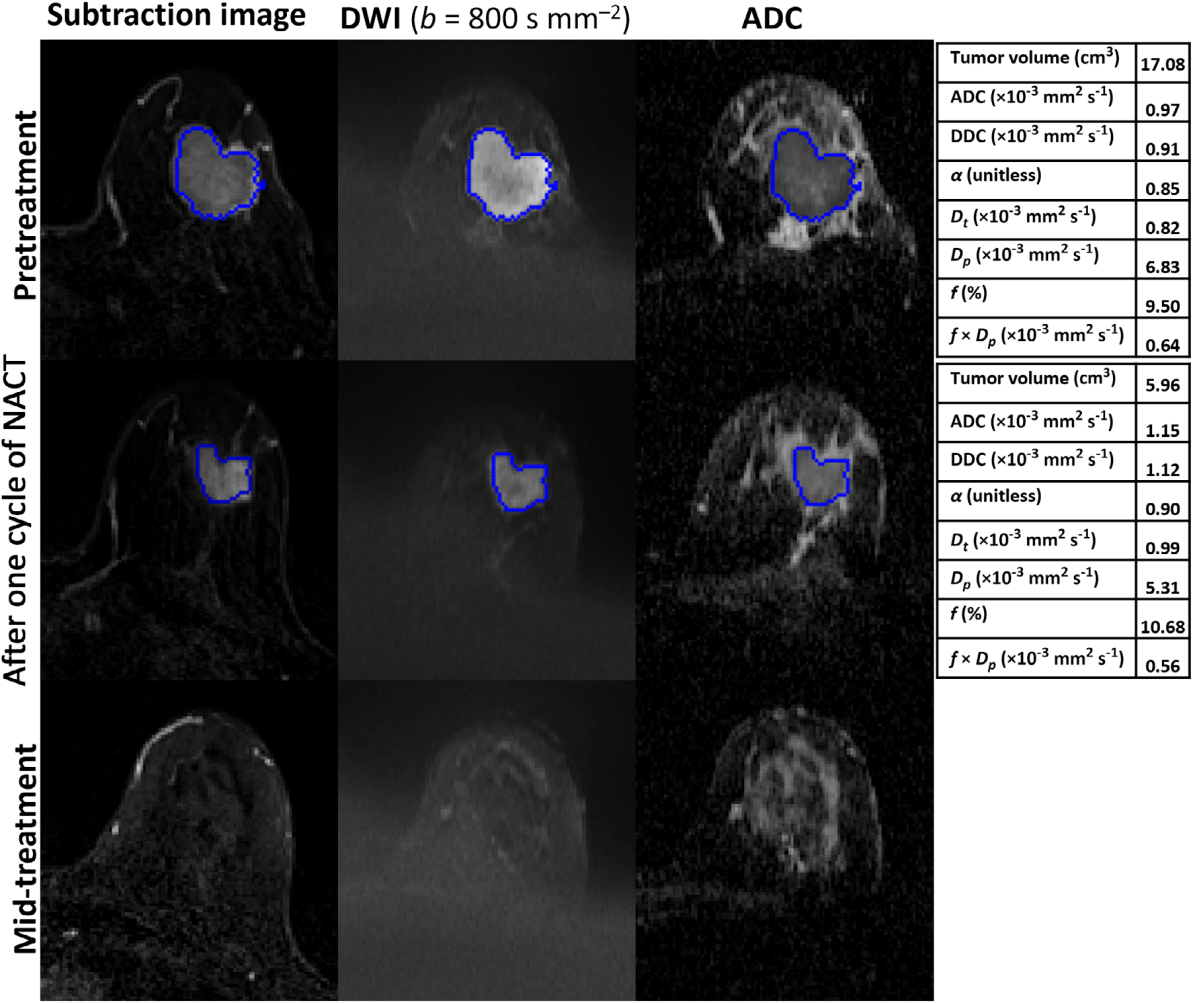
MRI scans of a 45‐year‐old woman with invasive ductal carcinoma in the left breast who showed a complete pathological response after surgery (residual cancer burden [RCB]‐0). Each row includes images acquired at pretreatment, after one cycle of neoadjuvant chemotherapy (NACT), and at mid‐treatment. The seeded region of interest (ROI) for the given slice is shown in blue. The tables represent the parameter estimates of monoexponential, stretched‐exponential model (SEM) and intravoxel incoherent motion (IVIM) model at each time‐point. At mid‐treatment, no tumor was visible on the dynamic contrast‐enhanced (DCE) and diffusion‐weighted (DW) images obtained.

Table 3 compares the parameter values of pR and pNR at pretreatment and after one and three cycles of NACT. At pretreatment and after one cycle of NACT, the analyses included 37 patients (pR = 17 and pNR = 20). However, two patients were excluded from the analyses at mid‐treatment because no tumor was visible in the MR images of these patients who went on to have a complete pathological response (Fig. 3) (pR = 15 and pNR = 20).

**TABLE 3:** Comparisons of Tumor Volume and Parameter Values at Pretreatment, After One Cycle of NACT, and at Mid‐treatment for the pR and pNR Groups

Parameter	Pretreatment (*n* = 37)			After One Cycle of NACT (*n* = 37)			After Three Cycles of NACT (*n* = 35)		
	pR	pNR	*P*	pR	pNR	*P*	pR	pNR	*P*
Tumor volume (cm^3^)	**2.03 (1.71, 4.65)**	**9.31 (4.64, 25.32)**	**<0.001**	**1.63 (0.87, 2.55)**	**8.78 (3.6, 24.24)**	**<0.001**	**0.52 (0.27, 1.87)**	**4.21 (1.96, 13.58)**	**<0.001**
ADC (×10^−3^ mm^2^ s^−1^)	0.98 (0.89, 1.06)	1.07 (0.91, 1.29)	0.40	1.04 (1, 1.14)	1.04 (0.97, 1.36)	0.84	1.19 (1.08, 1.31)	1.21 (1.05, 1.43)	1.0
DDC (×10^−3^ mm^2^ s^−1^)	0.93 (0.83, 1)	1.01 (0.82, 1.23)	0.42	1.01 (0.95, 1.1)	0.98 (0.88, 1.31)	0.94	1.12 (1.02, 1.3)	1.14 (0.99, 1.39)	0.85
*α* (unitless)	0.84 (0.8, 0.87)	0.82 (0.79, 0.86)	0.38	**0.89 (0.85, 0.92)**	**0.82 (0.78, 0.86)**	**0.015**	0.88 (0.8, 0.94)	0.84 (0.8, 0.87)	0.34
*D*_*t*_ (×10^−3^ mm^2^ s^−1^)	0.82 (0.78, 0.89)	0.84 (0.72, 1.02)	0.68	0.86 (0.84, 0.96)	0.83 (0.76, 1.06)	0.79	0.95 (0.87, 1.14)	0.97 (0.82, 1.13)	0.40
*D*_*p*_ (×10^−3^ mm^2^ s^−1^)	6.83 (5.27, 7.57)	6.47 (5.91, 7.28)	0.94	5.32 (4.07, 6.31)	6.44 (5.15, 7.61)	0.09	5.66 (3.96, 7.39)	6.04 (5.18, 8.67)	0.21
*f* (%)	11.13 (9.51, 14.26)	13.32 (10.94, 17.14)	0.09	**12.29 (10.22, 14.16)**	**16.21 (13.55, 18.1)**	**0.018**	14.38 (11.42, 16.45)	16.64 (14.44, 18.88)	0.21
*f* × *D*_*p*_ (×10^−3^ mm^2^ s^−1^)	0.84 (0.65, 0.95)	0.89 (0.71, 1.19)	0.21	**0.64 (0.52, 0.83)**	**1.08 (0.72, 1.24)**	**0.010**	0.89 (0.49, 1.18)	1.16 (0.76, 1.43)	0.09

Data are represented by medians (interquartile ranges). *P* value calculated using independent samples for the Mann–Whitney *U* test. Statistically significant results are highlighted in bold font.

NACT = neoadjuvant chemotherapy; ADC = apparent diffusion coefficient; DDC = distributed diffusion coefficient; *α* = diffusion heterogeneity index; *D*_*t*_ = tissue diffusion; *D*_*p*_ = pseudo‐diffusion coefficient; *f* = perfusion fraction; pR = pathological responders; pNR = pathological nonresponders.

Tumor volume for the pR group was significantly smaller than for the pNR group at all time‐points [median (cm^3^); pretreatment: pR = 2.03 and pNR = 9.31; post cycle one: pR = 1.63 and pNR = 8.78; post cycle 3, pR = 0.52 and pNR = 4.21). No significant differences were found in ADC, DDC, *D*_*t*_, and *D*_*p*_ between pR and pNR groups at all time‐points (pretreatment: *P* = 0.40, 0.42, 0.68, and 0.94; after cycle 1: *P* = 0.84, 0.94, 0.79, and 0.09; after cycle 3, *P* = 1, 0.85, 0.40, and 0.21, respectively). After one cycle of NACT, *α* values were significantly higher in the pR group (median; pR = 0.89 and pNR = 0.82). In contrast, pNR patients exhibited considerably higher *f* and *f* × *D*_*p*_ values [median: pR = 12.29 and pNR = 16.21 for *f* (%); pR = 0.64 and pNR = 1.08 for *f* × *D*_*p*_ (×10^−3^ mm^2^ s^−1^)]. However, no significant differences between the response groups were found in *α*, *f*, and *f* × *D*_*p*_ values after cycle 3 (*P* = 0.34, 0.21, and 0.09, respectively).

Table 4 summarizes the ROC curve analyses for all parameters. Tumor volume demonstrated reasonable accuracy in predicting treatment response at all time‐points (AUC = 0.83–0.87). In contrast, the AUCs for ADC, DDC, *D*_*t*_, and *D*_*p*_ demonstrated poor accuracy at all time‐points. After one cycle of NACT, response prediction was reasonable for *α* (AUC = 0.732), *f* (AUC = 0.726), and *f* × *D*_*p*_ (AUC = 0.744). At mid‐treatment, response prediction was poor for *α*, *f*, and *f* × *D*_*p*_ (AUC = 0.595, 0.628, and 0.670, respectively).

**TABLE 4:** Diagnostic Performance of Tumor Volume and Monoexponential, SEM, and IVIM Parameters in Predicting NACT Treatment Outcomes

Parameter	Pretreatment (*n* = 37)			After One Cycle of NACT (*n* = 37)			After Three Cycles of NACT (*n* = 35)		
	AUC	95% Confidence Interval	*P*	AUC	95% Confidence Interval	*P*	AUC	95% Confidence Interval	*P*
Tumor volume (cm^3^)	**0.838**	**0.707–0.969**	**<0.001**	**0.868**	**0.747–0.989**	**<0.001**	**0.872**	**0.756–0.987**	**<0.001**
ADC (×10^−3^ mm^2^ s^−1^)	0.582	0.393–0.772	0.39	0.521	0.326–0.715	0.83	0.503	0.307–0.699	0.97
DDC (×10^−3^ mm^2^ s^−1^)	0.579	0.391–0.768	0.41	0.508	0.312–0.706	0.92	0.520	0.325–0.715	0.84
*α* (unitless)	0.585	0.396–0.774	0.37	**0.732**	**0.564–0.901**	**0.01**	0.595	0.392–0.798	0.34
*D*_*t*_ (×10^−3^ mm^2^ s^−1^)	0.541	0.351–0.761	0.67	0.526	0.333–0.720	0.78	0.587	0.395–0.788	0.38
*D*_*p*_ (×10^−3^ mm^2^ s^−1^)	0.509	0.313–0.705	0.92	0.662	0.478–0.845	0.09	0.628	0.429–0.827	0.20
*f* (%)	0.664	0.488–0.842	0.08	**0.726**	**0.555–0.898**	**0.01**	0.628	0.424–0.832	0.20
*f* × *D*_*p*_ (×10^−3^ mm^2^ s^−1^)	0.620	0.438–0.803	0.21	**0.744**	**0.582–0.906**	**0.01**	0.670	0.481–0.859	0.09

Statistically significant results are highlighted in bold font. SEM = stretched‐exponential model; IVIM = intravoxel incoherent motion; NACT = neoadjuvant chemotherapy; AUC = Area under the receiver operating characteristic curve; ADC = apparent diffusion coefficient; DDC = distributed diffusion coefficient; *α* = diffusion heterogeneity index; *D*_*t*_ = tissue diffusion; *D*_*p*_ = pseudo‐diffusion coefficient; *f* = perfusion fraction.

There was no statistically significant relationship between the percentage change in the parameter values after one and three cycles of NACT and pathological response nor in the percentage change in tumor volume after one and three cycles of NACT and pathological response (After one cycle: *P* = 0.12–0.44; after three cycles: *P* = 0.06–0.58). The interobserver agreement (repeatability) in tumor volume, measured pretreatment, after one cycle of NACT, and after three cycles was excellent (ICC = 0.92, 0.98, and 0.99, respectively) (Table 5). A significant positive correlation was found between ADC, DDC, and *D*_*t*_ at all time‐points (*r* = 0.83–0.99) (Figure E1 in the Supplementary Material, ESM).

**TABLE 5:** Interobserver Agreement for the Tumor Volume Measured by Two Observers

	*n*	ICC (95% CI) “Absolute Agreement”	*F*	*P*
Tumor volume (at pretreatment)	37	0.919 [0.827, 0.960]	27.668	<0.001
Tumor volume (post one cycle)	37	0.976 [0.909, 0.991]	122.041	<0.001
Tumor volume (post three cycles)	35	0.990 [0.978, 0.995]	231.022	<0.001

ICC = intraclass correlation coefficient; CI = Confidence interval.

## Discussion

Early prediction and monitoring of the response to NACT are advantageous for individualizing an optimal treatment plan for a patient with breast cancer by avoiding exposure to ineffective NACT. This preliminary study examined predictions that could alter treatment early (i.e., by cycle 3 at the latest). The predictive power of monoexponential, SEM, and IVIM DWI models for determining NACT outcome for breast cancer in 37 patients was evaluated. The parameters of the DWI models were measured at three time‐points: pretreatment, after one cycle of NACT, and after three cycles.

The findings revealed that tumor volumes measured using the semiautomated method at the three time‐points for pR were significantly smaller than those for pNR, and tumor volume was able to predict response to NACT with reasonable accuracy. This confirms the finding shown in a recent study^28^ that tumor volume, measured using manually drawn ROIs, is a good predictor pretreatment and after one cycle of NACT. The present study showed that this prediction remains valid after three cycles. Moreover, the semiautomated method used in this study for tumor volume estimation has excellent repeatability.

A strong positive correlation of ADC with DDC and *D*_*t*_ was found at each MRI time‐point in the present study, suggesting that DDC and *D*_*t*_ can be interpreted in a similar manner as ADC in terms of observing diffusion components within the microenvironment. Similar to Surov et al,^32^ the results illustrated that none of the pretreatment diffusion coefficients predict response to NACT. Nonetheless, pNR in the present study had slightly higher pretreatment ADC, DDC, and *D*_*t*_ values than pR, as reported in previous studies.^20^, ^33^

After one cycle of NACT, no significant difference in ADC was noted between the response groups. This conflicts with the results reported by Li et al^34^ and may reflect: 1) technical differences in how ADC were calculated; 2) the use of different treatment regimens; and 3) how pathological response was evaluated.

Following one cycle of NACT, the percentage change in the tumor diffusion coefficients was not predictive of response, which is in line with a previous report.^27^ At mid‐treatment, no significant difference was observed between the response groups in the relative increase in ADC and DDC. This finding is inconsistent with the results reported by Bedair et al.^20^ In the present study, the ROIs were generated around the whole‐tumor volume on the DCE subtraction images, and the ROIs were then copied to the DWIs, and the average SI value for every *b*‐value was calculated. In contrast, Bedair et al generated parametric maps of all diffusion parameters, and then, the single‐section ROIs were analyzed on a voxel‐wise basis, and the parameters were expressed as means over the single‐section ROIs.^20^ Estimation of model parameters is more accurate when performed using the ROI averaged signals, compared to the average of parameter values estimated on a voxel‐by‐voxel basis.^26^ Moreover, volumetric sampling of the entire tumor may minimize sampling bias in comparison with the single‐section ROI method,^35^ and this method is recommended when evaluating tumor response.^25^ There were more low *b*‐values used in the present study. There were differences in the number of patients included in the mid‐treatment analysis and the method of categorizing response groups. In this study, 35 patients categorized as pR (15 patients, RCB‐0/I) and pNR (20 patients, RCB‐II/III) were included at the mid‐treatment analysis. In the study by Bedair et al,^20^ 22 patients were classified as pCR (8 patients) and non‐pCR (14 patients) at the mid‐therapy analysis. However, the ACRIN 6698 multicenter trial found that the percentage change in ADC value was predictive only in hormone receptor positive/HER2 tumors after four cycles of NACT.^27^

The values of *f* and *f* × *D*_*p*_ at the three time‐points were always higher in pNR compared to pR, and both were able to differentiate the two groups and predict response with reasonable accuracy after one cycle of NACT. Le Bihan et al state that the *f* parameter represents the blood volume fraction in a voxel, while *f* × *D*_*p*_ reflects blood flow.^21^ Thus, the higher *f* and *f* × *D*_*p*_ values in the pNR group may be attributed to the richer blood supply in the nonresponder tumors. Moreover, Lee et al found a significant positive correlation between *f* and histological microvessel density,^36^ a surrogate marker of tumor angiogenesis where high scores are often associated with poor prognosis after chemotherapy.^37^ A recent study also found that breast tumors with higher blood score by optical imaging were associated with poorer pathologic response to NACT.^38^ However, further investigation is required to determine the nature of the *f* and *f* × *D*_*p*_ parameters and their relationship to response to NACT.

Like Bedair et al,^20^ pR in the present study had higher *α* values than pNR at all time‐points. However, after one cycle of NACT, *α* was significantly higher in pR, which showed an ability to differentiate the two groups and predict response with reasonable accuracy. The biological interpretation of the heterogeneity index *α* is still under consideration, it could reflect the complexity of the tissue microstructure.^39^ High *α* values in pR tumors could be indicative of more structural homogeneity, while low *α* values observed in pNR tumors may be suggestive of a more heterogeneous microenvironment; vascular heterogeneity and the existence of microscopic necrosis, which results in a more aggressive tumor with less sensitivity to chemotherapy.^40^

## Limitations

First, the study was carried out in a single center using one scanner (1.5 T MRI; Aera; Siemens). Second, the sample size was small, which limits its interpretation. A subsequent study in multiple centers using different scanners with a larger sample cohort (responders and nonresponders) is recommended to validate the prediction performance of the DWI models. Third, the effects of voxel‐wise analysis and estimation of ADC using different *b*‐value combinations on the prediction performance were not investigated. Finally, the reproducibility of the monoexponential, SEM, and IVIM models' parameters was not examined. An upper estimate of the reproducibility of these diffusion models parameters was calculated, and the results were promising^9^ (Appendix E2, ESM).

## Conclusions

This preliminary study showed that analyzing the diffusion data with non‐monoexponential models provides better prediction of response to NACT than a monoexponential model. The IVIM‐derived parameters *f* and *f* × *D*_*p*_ and the SEM‐derived parameter *α* were able to predict response in patients with breast cancer with reasonable accuracy after one cycle of NACT. The results indicated that ADC, DDC, and *D*_*t*_ could not predict response pretreatment, after one cycle or three cycles. Tumor volumes in the responders were lower than nonresponders at all three time‐points. Patients who had a smaller tumor volume, a higher *α* value, and lower *f* fraction and *f* × *D*_*p*_ after one cycle of NACT were observed to respond better to NACT.

## Supporting information


**Appendix** E1: Supporting Information
