# Supplementary material for: Evaluation of Monoexponential, Stretched‐Exponential and Intravoxel Incoherent Motion MRI Diffusion Models in Early Response Monitoring to Neoadjuvant Chemotherapy in Patients With Breast Cancer—A Preliminary Study
Source: J Magn Reson Imaging. 2022 Feb 14;56(4):1079–88. doi: 10.1002/jmri.28113 (PMC9543625; doi:10.1002/jmri.28113)
Supplement: Supplementary file 1 — Appendix E1: Supporting Information [file JMRI-56-1079-s001.docx]

**ELECTRONIC SUPPLEMENTARY MATERIAL**

**Appendix E1**

**Diffusion models parameters estimation**

The parameter values of the monoexponential, SEM, and IVIM models were estimated using the solver function in Microsoft Excel 2010, where the SI versus b-value curves for each of the three models were fitted using the nonlinear least-squares method with the following mathematical models:

1. Monoexponential model:

$S\left( b \right)=S\left( 0 \right).\exp(-b .ADC)$,

where S(b) is the mean SI with diffusion weighting b, and S(0) is the mean SI obtained without diffusion weighting (b=0 s/mm^2^). S(0) and ADC value were simultaneously estimated from a monoexponential fit using the entire range of b-values. The ACRIN 6698 multicentre trial reported using signal intensities acquired at all b-values for ADC calculation (1).

1. SEM:

$$S\left( b \right)=S\left( 0 \right).{\exp(-((b . DDC)}^{\alpha})),$$

where DDC represents the mean intravoxel diffusion rate, and α is the heterogeneity index describing the deviation of the signal attenuation from monoexponential behavior with a value between 0 and 1. The entire range of b-values was used to provide the best-fit estimates for S(0), DDC, and α simultaneously.

1. Biexponential model (IVIM):

$S\left( b \right)= S\left( 0 \right). [\left( 1-ƒ \right)\exp\left( -b . Dt \right)+ ƒ \exp\left( -b . Dp \right)]$,

where f is the perfused fraction reflecting the fraction of pseudo-diffusion linked to microcirculation, Dt represents tissue diffusion, and Dp denotes the pseudo-diffusion coefficient. The Dt value was calculated first by linear least squares, using the logarithm of (SI) at b-values of 400 and 800 s/mm^2^. This calculation assumes that the Dp effect on the signal is negligible when the b-value ≥400 s/mm^2^ (2). Subsequently, S(0), Dp, and f were estimated from a biexponential fit with a fixed Dt value (obtained above) using all b-values.

**Figure E1.** Scatter plots for the diffusion coefficients show the significant relationship between the ADC, DDC, and Dt parameters. The correlation coefficients (r-values) were obtained from the Spearman correlation tests, and the corresponding P-values were annotated for each plot.


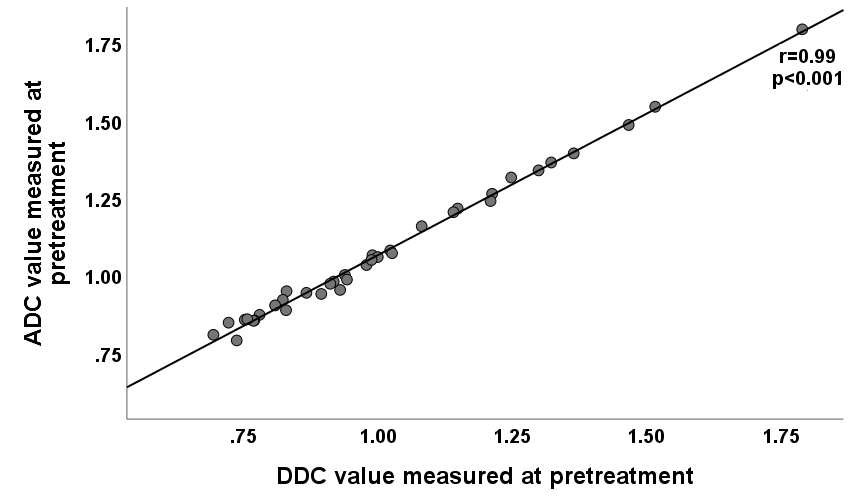

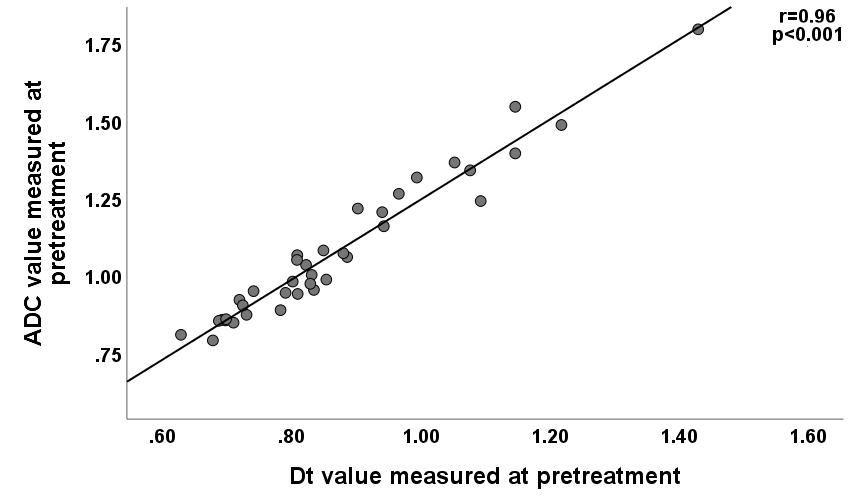

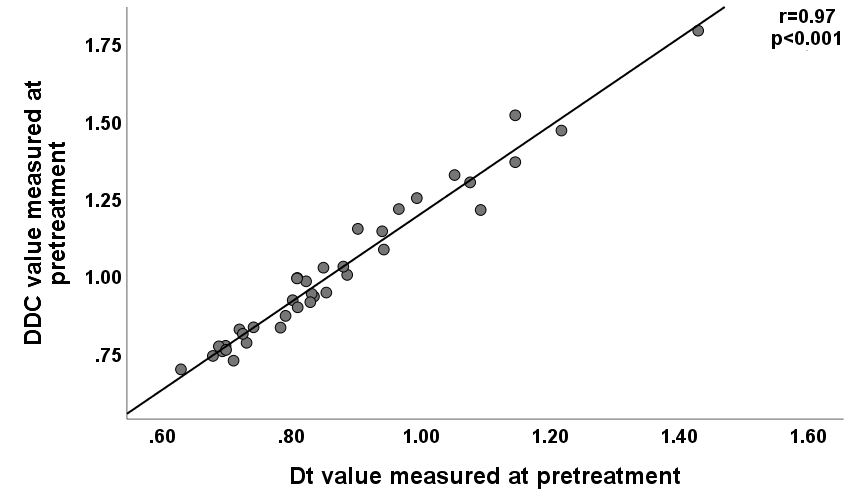

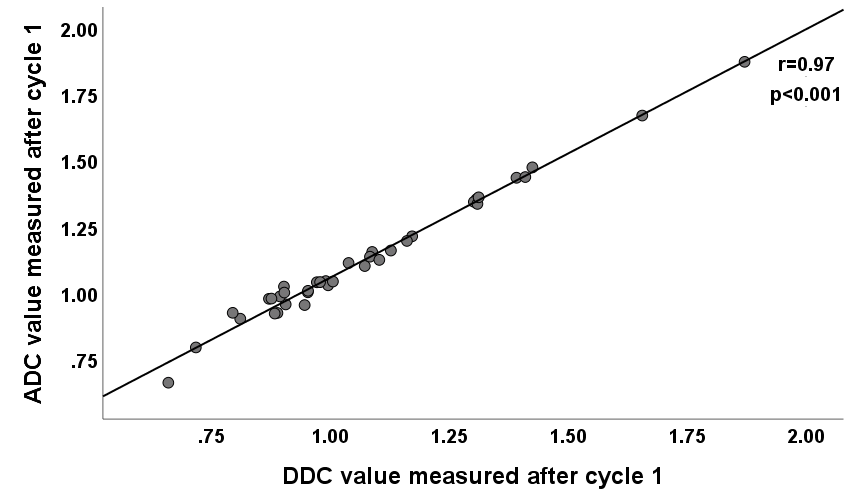

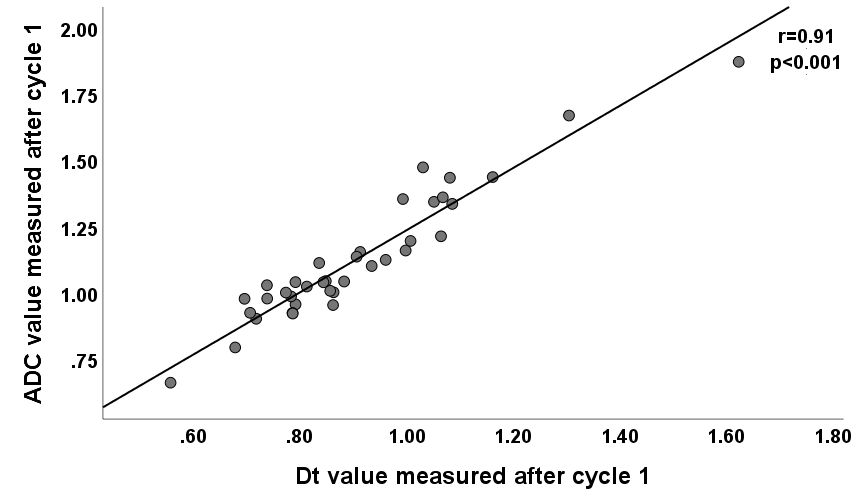

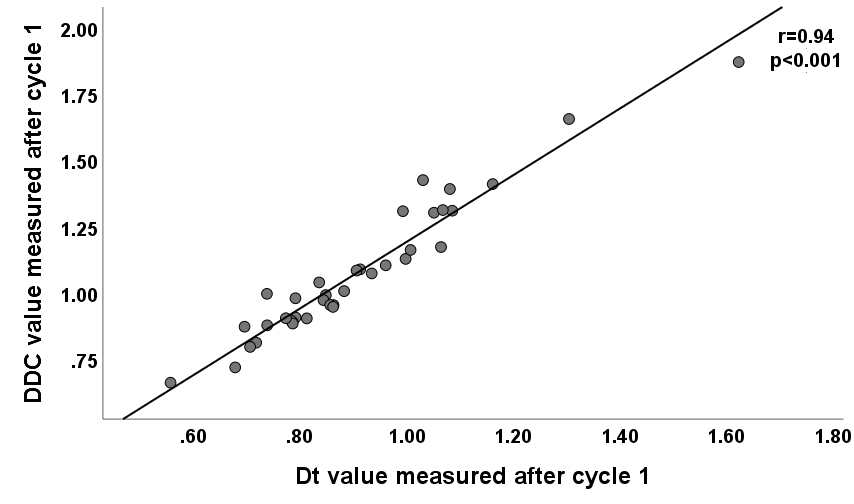

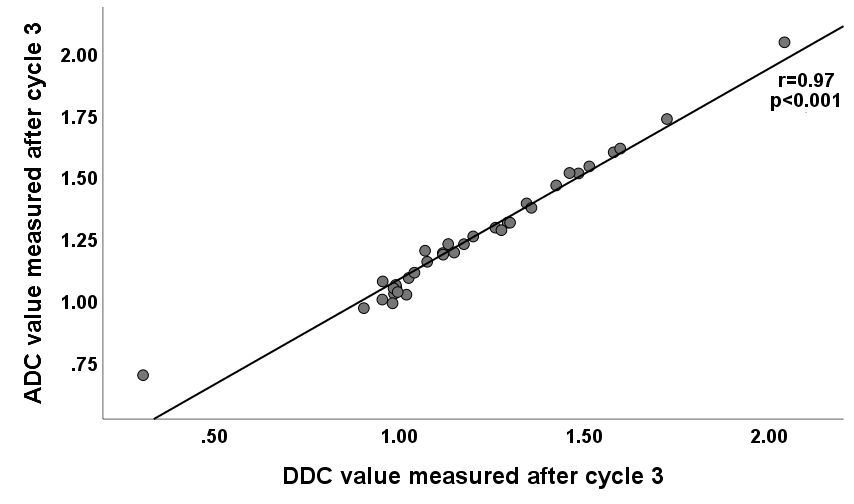

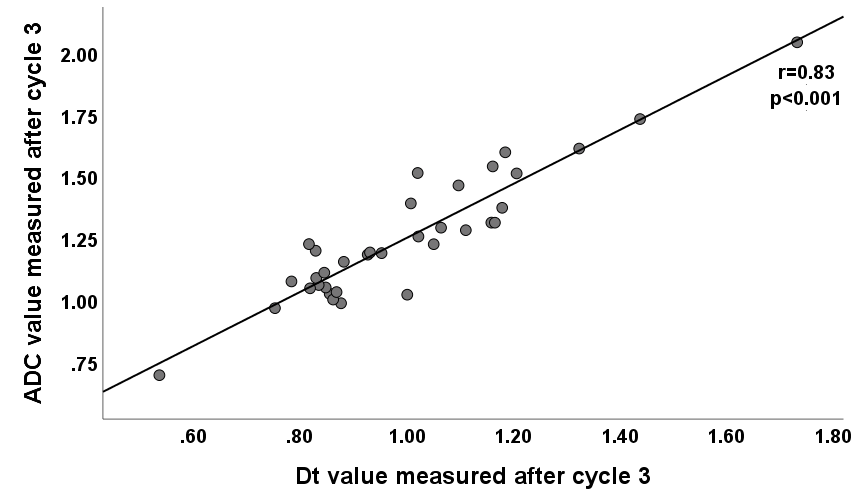

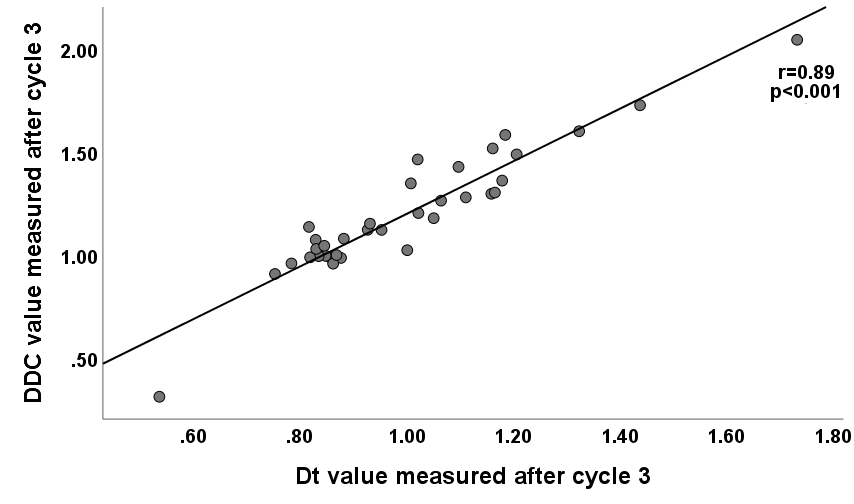


**Appendix E2**

**Reproducibility analysis and results**

An upper estimate of the reproducibility of the diffusion model parameters was assessed by calculating the within-subject coefficient of variation (wCV) (3) in the pretreatment and post-one cycle data of a subset of pNR who showed the smallest changes in tumor volume at cycle 1. We chose pNR patients with tumor volume changes between -66% (shrinkage) and +73% (increase). It is indicated that the tumor volume changes would have to shrink by -66% to be considered a partial responder or increase by +73% to be considered a progressive disease (4).

After one cycle of NACT, 10 pNR were included in the reproducibility analysis. The 10 tumors showed changes in volume between -17.80% (shrinkage) and +59.40% (increase), with an average volume change of +10.35%. The upper estimate of the wCV for ADC was 5.9%; for the SEM parameters, it was 7.1% for DDC and 4% for α; for the IVIM parameters, it was 6.9% for Dt, 14.5% for Dp, 8.3% for f, and 19.2% for f*Dp.

**References:**

1. Partridge SC, Zhang Z, Newitt DC, et al. Diffusion-weighted MRI findings predict pathologic response in neoadjuvant treatment of breast cancer: the ACRIN 6698 multicenter trial. Radiology 2018;289(3):618-627.

2. Bedair R, Priest A, Patterson A, et al. Assessment of early treatment response to neoadjuvant chemotherapy in breast cancer using non-mono-exponential diffusion models: a feasibility study comparing the baseline and mid-treatment MRI examinations. Eur Radiol 2017;27(7):2726-2736.

3. Shukla‐Dave A, Obuchowski NA, Chenevert TL, et al. Quantitative imaging biomarkers alliance (QIBA) recommendations for improved precision of DWI and DCE‐MRI derived biomarkers in multicenter oncology trials. J Magn Reson Imaging 2019;49(7):e101-e121.

4. Eisenhauer EA, Therasse P, Bogaerts J, et al. New response evaluation criteria in solid tumours: revised RECIST guideline (version 1.1). Eur J Cancer 2009;45(2):228-247.
